# The Gap in Community Sports: Utilization of Sports Facilities in South Korea

**DOI:** 10.3390/ijerph19084495

**Published:** 2022-04-08

**Authors:** Minuk Kang, Youngjik Lee

**Affiliations:** Department of Physical Education, Kookmin University, Seoul 02707, Korea; minuk@kookmin.ac.kr

**Keywords:** local community, sports facilities, inequality, sports welfare, South Korea

## Abstract

Anyone can participate in sports, but not everyone has the opportunity to do so. The purpose of this study was to identify the factors causing inequality in sports participation based on the causes of the disparity in sports facility utilization in South Korea. Based on ecosystem theory, factors affecting the use of sports facilities were identified. For the causal relationship analysis of each factor, a hierarchical regression analysis was performed using the SPSS (version 26.0) package (IBM, Armonk, NY, USA). The characteristics of individual, family, and community levels show the different impacts based on study models with hierarchical structures. The results of this study illustrated that family characteristics did not influence the facilities’ utilization rate. However, individual and community characteristics did influence the sports facilities’ utilization rate. Although these results were derived from the case of South Korea, they are comparable data focusing on country-specific characteristics and community indicators. It is expected that sports participation can be strengthened by bridging the gap in sports facilities’ utilization.

## 1. Introduction

Inequality can cause many social problems and may lead to the repetition of a vicious cycle, entailing social costs to resolve and cure social problems. This social pathological phenomenon is a cost that all members of a society should bear. Thus, devising solutions for the risk factors is a necessary task to be fulfilled [1]. Furthermore, the problem becomes more severe because the inequality prevailing in society raises the problem of poverty and could hinder the balance of interdependence and trust, which are the foundations of civil society [2]. This reminds us why we should pay attention to the efforts to redress the unequal social environment to create a healthy society.

One of the representative scholars in the field of social welfare, Esping-Andersen, highlighted in his book *The Incomplete Revolution* in 2009 that the level of threats to our society by the inequality of wealth and the social injustices experienced by people in the process of the development of modern society is ascending. In particular, he outlined the magnitude of the problem that social inequality from income imbalance gives rise to social polarization and causes risks for the most vulnerable class [3]. Furthermore, Richard Wilkinson, an English researcher in the field of social epidemiology, in his book *Unhealthy Societies, the Afflictions of Inequality* in 1996, suggested the Wilkinson hypothesis, which explains the influence of income inequality on socioeconomic factors, including health. This theory emphasized the negative impact of the increasing level of income inequality on public health and investigated the influence of the levels and types of income on the health of individuals based on a causal relationship model [4,5]. Economic polarization could be suggested as one of the major contributors to inequality; however, what influences the health of individuals and society is not just limited to economic factors but is gradually expanding to differences at the individual and regional levels [6,7].

The problems caused by this inequality have been occurring in sports. Various sports participation opportunities arise along with increasing social interests in health and the development of markets in the sports industry; however, the disparities due to socioeconomic or cultural factors lead to the poverty of sociocultural capital. With recognizing sports participation as a basic right of the people, the government has established policy-level systems, including expansion of programs, instructors, facilities, and information to activate sports participation.

Based on the comprehensive national sports promotion plan in Korea, the three essential components of daily sports can be divided into programs, instructors, and facilities. Programs and instructors could be classified as human resources and facilities as material resources. Reviewing the related preceding studies, research such as surveys on the actual condition and methods for activation of daily sports participation tends to focus mainly on the administrative and human resources [8,9]. Based on this research trend, the suggested problems are the lack of scholarly attention to sports facilities and the lack of academic recognition of dealing with sports facilities from a sports welfare perspective. To search for solutions to achieve the efficiency and effectiveness of daily sports, an approach focusing on a balanced perspective is required for the three components of daily sports, namely, programs, instructors, and facilities [9].

Ecosystem theory explains a social phenomenon not through a single factor but through a multidimensional perspective. In other words, it is an effective theory enabling the analysis of interactions between environmental variables focusing on the constant reciprocal relationships with the environment surrounding individuals on the basis of “person in environment” [10]. This ecosystem theory is a concept in which one analyzes and understands the relationships between individuals and environments and is understood to be a theory to expand the scope of understanding the research subject and investigate the cause for certain behaviors [11,12,13].

The gap, in general, means differences. It encompasses differences according to institutions and lifestyles as well as whether goods are owned or not. In this context, the gap in sport means differences in not only individuals’ physical ability or spontaneous will but also in participation opportunities for daily sport by social environmental factors. The factors that influence individuals’ health include not only biological factors such as sex, ethnicity, and physical composition, but also sociological factors including social class, residential environment, and so on. Thus, considering the dynamics from an ecological perspective is suggested as a proper way to understand the gaps of relative and multidimensional concepts formed by the interactions of individuals and societies [12,14].

The factors influencing the sports activities of individuals has been classified into stages such as individual, interpersonal, environmental, regional or national policy, and global relation [15]. The factors influencing the decisions for sports activity participation were divided into actions at individual levels, including interpersonal actions through psychological and biological factors, and actions at interpersonal relationship levels, including social support and sociocultural norms influenced by other people [15]. The environmental context was divided into the social environment, such as relationships with surrounding people and various events occurring from social phenomenon, the cultivated environment such as group communities and regional facilities, and the natural environment such as forests, parks, weather, and so on. Moreover, national policies, being a superordinate concept, were composed of organized and systemic activities, such as designed city environments, campaigns, etc., and were expanded to the stage of internationalization, such as economic growth, interests in international society, and so on.

This study mainly deals with the analysis of the cause of the gap in the utilization of sports facilities to analyze the factors causing inequality in daily sports participation and offers proof to alleviate this inequality. This begins with problem recognition to prove the distinctive characteristics of sports facility environments from the users’ perspective. Exploring the regional characteristics and the gap in sports through the case of Korea is academically meaningful in view of pursuing the sustainable growth of global phenomena such as urbanization and localization. The research question investigates the differences in the use of sports facilities according to individual, family, and community characteristics.

## 2. Materials and Methods

### 2.1. Data Collection

To analyze the gap in sports facilities’ utilization, the utilization rate of sports facilities was set as the dependent variable, and a study model was designed to investigate the causation between the variables influencing it. Domestic residents were selected as the study subjects, and the daily sports participation rate was investigated with the use of data on whether or not sports facilities were used and how often they were used. Based on the study purpose and the population, the actual state of sports facilities’ utilization and major indexes of community units were selected as the study subjects. For the secondary data, the National Sports Participation Survey announced by the Ministry of Culture, Sports, and Tourism, and the Community Health Survey announced by the Centers for Disease Control and Prevention, an affiliated organization of the Ministry of Health and Welfare, as well as the data on the number of people and gross regional income by administrative district from the Korean Statistical Information Service (KOSIS), were collected.

The National Sports Participation Survey was performed for the first time in 1986 in pursuit of improving the daily sports participation environment and drawing policy-level data by surveying the actual state of sports activities of the nation and resolving factors restricting participation and was based on Article 33 of the Statistics Act, the State-approved designated statistics No. 11303. In addition, a Community Health Survey was performed in pursuit of producing regional health statistics for establishing and evaluating regional healthcare and medical plans and was based on Article 33 of the Statistics Act, the State-approved designated statistics No. 11775. KOSIS provides various data generated by public organizations based on years or subjects. The secondary data collected as above were generated by state organizations and distributed to the public, thereby verifying public confidence. Furthermore, with consideration of the representative nature, sampling method, and item suitability of the data, a high level of reliability and validity was guaranteed. Thus, the selected variables, such as the National Sports Participation Survey and Community Health Survey, were judged as proper data to fulfill the study purpose. In particular, it is expected that study results utilizing the secondary data selected for study variables considering the distinct characteristics of the state-approved statistical data, in which the actual condition of sports participation is measured on a national scale, and the scarcity of macro-indicators in the field of sports, could suggest academic and policy-level implications.

To match the content and survey period among the secondary data collected, the data from the most recent year available were collected. The National Sports Participation Survey was performed from September to November 2020, and the results were opened to the public in January 2021. In addition, the Community Health Survey proceeded from August to October 2020. For the data on the number of people and gross regional income by administrative district, the content of the final announcement by the National Statistical Office in December 2020 was applied. For the secondary data collected as above, measures were taken to guarantee the homogeneity of the data by matching survey periods; hence, each data point input as a community variable can be understood as properly applied regional characteristics. To estimate the sports facilities’ utilization rate, models were classified based on the content of responses to questions on whether or not sports facilities were used. Each community variable was matched based on residents by administrative districts. The total number of sample cases used the data of 9012 persons.

### 2.2. Variables

The “sports facilities’ utilization rate”, a dependent variable used for the analysis of the gap in sports facilities’ utilization, was calculated based on the number of participants in daily sports using sports facilities in comparison with the standards based on periods. Specifically, the responses on the type and utilization frequency (number of utilizations and week/month/year) of frequently used sports facilities were converted into ratio per period and ratio per unit to estimate the degree of sports facilities’ utilization. In the “sports facilities’ utilization rate”, the higher the utilization of sports facilities within the unit period, the higher the points. Thus, it can be interpreted as follows: the higher the utilization rate of sports facilities, the more active the daily sports participation is in these sports facilities.

The independent variables influencing the dependent variables were selected in consideration of not only individual levels but also societal structural levels. The gap in sports facilities’ utilization was analyzed focusing on the investigation of causal pathways between variables based on individual characteristics and social environmental characteristics by approaching from the perspectives of ecosystem theory and environmental epidemiology. A three-phased hierarchal structure was composed specifically on the basis of the ecological level being a multidimensional concept. Level 1 is composed of individual characteristics such as “sex”, “age”, “subjective health perception”, and so on. Level 2 comprises family characteristics such as “the number of family members”, “monthly family income”, and so on. Lastly, Level 3 consists of community characteristics such as “urban scale”, “quality-of-life index”, “yearly utilization rate of health organization”, “regional gross income per capita”, and so on.

Among the composed research variables, in the case of categorical data, some parts could not be statistically processed by hierarchal regression analysis due to the characteristics of nominal scale; therefore, they were converted into dummy variables that could be used in the regression analysis. Specifically, among the Level 1 individual characteristic variables, “sex” was divided into male and female respondents, converted into 0 for male and 1 for female. In the case of the Level 2 family characteristic variables, “the number of family members” and “monthly family income” were continuous data in which no extra conversion was required. Meanwhile, among the community variables, “urban scale” and “type of sports facilities” were processed according to the statistical process with the standard value of 0, and other responses were converted into the value of 1. “Urban scale” differed depending on the population of each region. Considering the circumstances in Korea, cities with a population over 1 million were classified as large cities, and cities with a population of less than 1 million were classified as small cities or farming areas.

The Korea Centers for Disease Control and Prevention aggregated the quality-of-life index, called the EQ-5D (EuroQol five dimension scale) index, where five kinds of quality-of-life technical systems related to health were collected, including exercise capacity, self-control, daily activities, pain/discomfort, and anxiety/depression. In the quality-of-life index, the lower the Likert scale, the lower the quality of life. The yearly utilization rate of healthcare organizations is the index that shows the number of people who have used healthcare organizations for the recent year. Healthcare organization means all of the public health centers (health center and county hospital), the branch office of the community health center, and the healthcare center. For the questions about the utilization of healthcare organizations, the results show the number of respondents converted into a percentage [16]. That is, a higher response rate means more experiences of healthcare organization utilization and is an indicator from which inferences about the level of exposures to diseases and the health of the population residing in related regions can be made.

### 2.3. Data Analysis

This study attempted to analyze the gap in sports facilities’ utilization using empirical data. In addition, the study investigated the reasons for the occurrence of the gap in sports facilities’ utilization from an ecological perspective. It structured the independent variables in a hierarchical form, where sports participation was categorized into various levels, including individual, family, and community levels, rather than limiting it only to the free will of individuals. To analyze causation according to the characteristics of these variables, hierarchical regression analysis was selected rather than multiple regression analysis.

Hierarchical regression analysis is a method using a multilevel model that structures independent variables into various levels based on the results of previous studies. Thus, by controlling or adding the effects of theoretically separated independent variables, this study compares the influence of independent variables on dependent variables and determines the explanatory power and the degree of contribution of the regression analysis model. Independent variables are composed according to the level of each phase based on the study design, reflecting the hierarchical or intrinsic characteristics of the data. Thus, hierarchical regression analysis is differentiated from multiple regression analysis in the sense that the influence of each independent variable on the dependent variables can be compared and measured. Specifically, in the case of multiple regression analysis, it is difficult to satisfy the assumptions of the independence and homoscedasticity of variables, because the multidimensionality between independent variables is not considered, and concordance estimates of regression coefficients cannot be expected. Meanwhile, hierarchical regression analysis can explain the influence of each hierarchical level based on the significance between the variables, thus guaranteeing independence between the variables.

The Statistical Package for the Social Sciences (SPSS version 26.0) was used to perform hierarchical regression analysis. For all the independent variables of various levels, an option method called the “input” method was selected, and the significance level was set at 0.05. To verify multicollinearity among independent variables, the basic premise of regression analysis, the values of tolerance and the variance inflation factor (VIF) were set as the standard. The multicollinearity could be verified with the correlation value as well, but this cannot be used when the independent variable is of bivariate normal distribution; moreover, when the independent variable does not satisfy normality or has an abnormal value, the resulting value could be problematic. Thus, the multicollinearity between independent variables was verified through the values of tolerance and VIF. The tolerance of the independent variables input into the three-level hierarchical regression equation where community characteristics were added was found to be lower than 1.0, and the VIF was lower than 5.0. Thus, there was no multicollinearity among the variables.

## 3. Results

### 3.1. Sports Facilities’ Utilization Based on Individual Characteristics

The results of the Level 1 hierarchical regression equation analysis (see Table 1) consisting of individual characteristics such as “sex”, “age”, and “subjective health perception” revealed that only “age” and “subjective health perception” significantly influenced the “sports facilities’ utilization rate”. Specifically, it was found that the higher the “age (b = 0.320)”, the higher the “sports facilities’ utilization rate”, as well as the higher the “subjective health perception (b = 1.114)”, the higher the “sports facilities’ utilization rate”. Meanwhile, it was found that “sex (b = 0.236)” did not significantly affect the “sports facilities’ utilization rate”.

As a result of analyzing the influence of the independent variables on the dependent variables in the hierarchical regression equation, variables with a high β value were found to be in the order of “age (β = 0.202 ***)” and “subjective health perception (β = 0.148 **)”. As a result of the Level 1 hierarchical regression analysis consisting of individual characteristics, it was found that “age” influenced the “sports facilities’ utilization rate” the most, followed by “subjective health perception”.

### 3.2. Sports Facilities’ Utilization Based on Family Characteristics

The results of the Level 2 hierarchical regression equation analysis (see Table 1) consisting of family characteristics such as “the number of family members” and “monthly income of family” revealed that only the existing Level 1 independent variables of “age” and “subjective health perception” significantly influenced the “sports facilities’ utilization rate”. Specifically, it was found that the higher the “age (b = 0.304)”, the higher the “sports facilities’ utilization rate”, as well as the higher the “subjective health perception (b = 1.487)”, the higher the “sports facilities’ utilization rate”. Meanwhile, it was found that “the number of family members (b = 0.042)” and the “monthly income of family (b = 0.447)” did not significantly influence the “sports facilities’ utilization rate”.

As a result of analyzing the influence of the independent variables on the dependent variables in the hierarchical regression equation, the variables with the highest β values were determined to be “subjective health perception (β = 0.198***)” followed by “age (β = 0.148**)”. As a result of the Level 2 hierarchical regression analysis to which family characteristics were added, it was found that “subjective health perception” influenced the “sports facilities’ utilization rate” the most. The explanatory power of the regression equation (*R*^2^ = 0.224) where the independent variables explain the dependent variables was found to be 22.4%. The changes in the explanatory power of the hierarchical model (Δ*R*^2^ = 0.015) were found to be 1.5%, but this had no statistical significance. Compared with the Level 1 hierarchical regression analysis of individual characteristics, no statistical changes were shown in the case of the Level 2 hierarchical regression analysis where family characteristics were added.

### 3.3. Sports Facilities’ Utilization Based on Community Characteristics

The results of the Level 3 hierarchical regression equation analysis (see Table 1) consisting of community characteristics such as “urban scale”, “quality-of-life index”, “yearly utilization rate of healthcare organization”, and “regional gross income per capita” revealed that the above Level 3 independent variables and the Level 1 independent variables of “sex”, “age”, and “subjective health perception” significantly influenced the “sports facilities’ utilization rate”. Specifically, among the Level 3 community characteristics, in the case of “urban scale (small-sized cities, b = −0.388, countryside, b = −0.623)” compared with residing in large cities, those people residing in smaller cities had a lower “sports facilities’ utilization rate”. In other words, as a result of the hierarchical regression analysis converted into dummy variables, it was found that groups residing in large cities utilized sports facilities at a higher rate compared with those living in small- and medium-sized cities or farming areas. Moreover, it was apparent that the higher the “regional gross income per capita (b = 1.760)” and “quality-of-life index (b = 5.218)”, the higher the “sports facilities’ utilization”.

As a result of standardizing and analyzing the influence of the independent variables on the dependent variables in the hierarchical regression equation, the variable with the highest β value was determined to be “regional gross income per capita (β = 0.648 ***)”, followed by “quality-of-life index (β = 0.482 ***)”, “subjective health perception (β = 0.168 ***)”, “age (β = 0.146 ***)”, “urban scale (small-sized cities, β = −0.072 **, countryside, β = −0.179 ***)”, and “sex (female) (β = 0.008 *)”. Thus, as a result of the Level 3 hierarchical regression analysis to which community characteristics were added, it was found that “regional gross income per capita” influenced the “sports facilities’ utilization rate” the most. The explanation power of the regression equation (*R*^2^ = 0.717), where the independent variables explained the dependent variables, was found to be 71.7%. In the case of the Level 3 hierarchical regression analysis to which community characteristics were added, compared with the Level 2 hierarchical regression analysis of family characteristics, the change in explanatory power (Δ*R*^2^ = 0.507), where independent variables explained the dependent variables, was found to be increased by 50.7%. 

## 4. Discussion

This study attempted to investigate the causes of the occurrence of gaps in sports facilities’ utilization. In particular, the basis of the phenomenon where gaps in sports facilities’ utilization occur was suggested, and specific ways were offered to realize sports welfare by addressing sports facilities’ utilization gaps. Specific factors influencing the sports facilities’ utilization rate were analyzed, with the classification of the characteristics of individual, family, and community into various stages based on ecosystem theory.

The results determined by this study are as follows. First, among individual characteristics, only “age” and “subjective health perception” were found to have a significant influence on sports facilities’ utilization. Specifically, respondents who were older and reported to be in good health condition showed a high rate of sports facilities’ utilization. On the other hand, “sex” was found not to influence the sports facilities’ utilization rate. The high correlation between “subjective health perception” and sport participation is not new. Physical activity is effective for psychological well-being. This also plays a central role in determining self-perceived health. In other words, sport participation and perceived health interact positively [17,18]. However, there may be some differences in “age” and “sex”. According to a study on physical activity barriers for the older adult, the older the elderly, the more they face barriers due to lack of interest in health or lack of opportunities such as activities or transportation [19]. In general, physical activity is far more related among males. [19,20]. Differences in contrast to the results of this study are converged by applying the entire life cycle, not a specific subject. For groups of adults or seniors, it is understood that conditions for daily sports participation are ensured according to the perceived importance of healthcare and stability in their living situation. Nowadays, the daily sports participation gap between males and females has decreased, but problems could still exist in the limited participation in daily sports based on male/female sexes. Thus, to increase the sports facilities’ utilization rate in relation to individual characteristics, participation from younger ages should be encouraged as well, and where individuals’ perceived health condition is low, the fact that health promotion can be pursued through daily sports participation should be actively disseminated. The factors influencing individuals’ health are produced and reproduced by the interactions of biological characteristics such as sex, race, and social conditions. The use of sports facilities indicates not only the promotion of individuals’ health but also the societal culture promoting wellbeing and increased interest in the quality of life [5,8]. This suggests that as people age, they are engaged in more social activities, and they obtain relatively more significant influences from their surrounding environment and other people. The spontaneous will of individuals to pursue healthy lifestyles using sports facilities can be recognized as a major factor influencing the use of sports facilities.

Second, “the number of family members” and “monthly income of family” comprising the family characteristics were found to have no significant influence on the sports facilities’ utilization rate. Meanwhile, based on the characteristics of the hierarchical independent variables, “age” and “subjective health perception” were found to still influence the sports facilities’ utilization rate. The results demonstrated that individual characteristics had more of an influence than family characteristics. Specifically, these results reflect the increasing desire of individuals for healthy and prosperous lives and are highly related to increased interests and participation in daily sports. However, this does not mean underestimating the importance of family characteristics. Previous research suggested that family, brothers, and sisters can influence the decision making of individuals. From the social ecology perspective, the family functions as a structural frame of the community transformation process and serve as a service provision counselor. [10]. Bronfenbrenner’s social ecology model focuses on the progressive accommodation between the growing human organism and the changing environments. The relation between person and environment is conceived in systems [12]. Parents are the ones who influence their children’s participation in sports. If parents are involved in sports, so will their children, and they have a wide range of opportunities to join the club [21,22,23]. However, the results drawn from this study model suggest that family does not influence sports participation. Although there could be differences caused by various variables, such as the type of facility used and the participated sport, the results of this study are significant in that individual characteristics are suggested as an important variable influencing the decision to use sports facilities.

Third, all of the community characteristics of “urban scale”, “quality-of-life index”, “and regional gross income per capita” were found to significantly influence the sports facilities’ utilization rate. Specifically, subjects residing in large cities showed a high rate of sports facilities’ utilization, and so did people in the regions where the quality of life or regional gross income per capita were higher. The community characteristics were found to have the most significant influence on the sports facilities’ utilization rate compared with individual or family characteristics. This means that community characteristics are not limited merely to physical spaces for residence but also function as social spaces that impact individuals’ decision making. In particular, the sports facilities’ utilization rate of each region showed large differences based on regional gross income per capita. Specifically, it could be inferred that the higher the regional income, the higher the sports facilities’ utilization rate and daily sports participation rate, and this can be offered as a basis for the occurrence of sports gaps due to the inequality and imbalance of regional wealth. Lack of community-based programming causes negative impacts on youth sports participation and even the returning after subsequent injury treatment. The difference is evident in the shift from school-based sports participation to private club sports in the US [24]. Unhealthy behaviors such as smoking and poor nutrition according to socioeconomic status are directly related to a lack of physical activity. It means more than the inability to purchase goods and services that promote health [25]. In addition to sports and health, communities significantly impact disparities, including differences in education, medical service, housing, information, and food access [26,27,28]. Regions are restricted to physical spaces, and a region’s benefits reach only those who reside in that space. The utilization rate could vary based on these qualitative and quantitative differences in the levels of public facilities of various regions; thus, there could be gaps in the degree of activity participation as relates to poverty as well [16]. The characteristics of sports facilities being public goods could act as a basis to induce the daily sports participation of the citizens of communities, and this could be interpreted as being influenced by community characteristics such as urban scale, quality-of-life index, the yearly utilization rate of healthcare organization, regional gross income, and so on. Thus, in the case of sports facilities, user satisfaction should be increased through providing differentiated services by understanding the users’ demands.

Reviewing previous studies analyzing various factors influencing health from an ecological perspective, it has been reported from regional gaps that the standard death rate is the lowest in large cities but increases in the order of small- and middle-sized cities, agricultural regions, and coastal areas. Furthermore, the health condition of senior citizens residing in the capital area was different from that of those residing in non-capital areas. Higher income levels mean the formation of healthy daily habits for health enhancement, including giving up smoking, exercising, and so on; however, lower income levels mean exposure to activities disrupting health, including smoking, drinking, and irregular eating habits [4,6,7]. Such differences can be understood as factors causing an imbalance and inequality of social opportunities, resources, and power in the regional context.

In terms of social welfare, improving personal and social health through improving the sports environment has already been proven. Sports facilities, equipment, and the distribution of sports programs projects support developing countries. Projects in connection with the community positively contribute to public health [29]. A large-scale professional sports facility can have economic effects, such as revitalizing the local economy and the possibility of urban redevelopment and a balanced development effect through improving local welfare and urban redevelopment [30,31].

According to the case of Denmark and Norway, where the number of sports facilities per capita is high, there is a close relationship between welfare policy and the supply of sports facilities. Guaranteeing access to sports facilities is the basis of sports welfare policy. The more underdeveloped urban development is, the more government-led sports facilities are supplied [32]. Government policies, such as the provision of sports facilities through the formation of public and private partnerships, are ultimately returned to the community’s happiness.

Public health issues related to the well-being of residents can be approached from an ecological perspective [33]. It is unreasonable to view the utilization of sports facilities as sports and physical activity participation. However, when recognizing sports facilities as environmental conditions for sports participation, this is a factor that can resolve inequality in sports participation. In particular, the fact that the influence on the use of sports facilities is relatively high in the community compared to individuals and families presents a task for realizing sports welfare. Also, the environmental changes and contraction caused by COVID-19 are applied to sports too. The limited use and temporary closure of public and private sports facilities threaten the utilization of sports facilities. It is the same phenomenon not only in Korea but also worldwide. Since the research results are based on 2020, it is limited to drawing comparison results between the pre, mid, and post corona periods. Nevertheless, diseases that threaten public health, such as COVID-19, are considered to harm sports facilities in terms of environmental threats. It is necessary to observe environmental changes through longitudinal studies.

## 5. Conclusions

This study drew the conclusion that community characteristics are the mechanism causing gaps in sports participation between regions. The results of the hierarchical regression analysis of the effects of the individual, family, and community on the use of sports facilities based on ecosystem theory were as follows: The use of sports facilities significantly changed according to community factors rather than individual and family factors. This means that the higher the level of development of the community, the higher the participation in sports, such as the use of sports facilities. However, the use of sports facilities does not necessarily mean participation in sports, since there are sports that can be pursued without using a sports facility. Nevertheless, it is meaningful in terms of the government-centered sports policy promotion, as the main task is to create infrastructure including the supply of sports facilities to activate sports participation.

Based on the sports facilities’ utilization rate according to the qualitative and quantitative differences of sports facilities provided in communities, the realization of sports welfare should be approached through the efforts to minimize the sports gaps that could occur due to the differences between regions and social classes. Individual, family, and social characteristics cannot be defined as one single factor but are complex factors that are mutually influenced. Individual (sex, age, and subjective perception of health), family (number of family members and/or monthly family income), and community (urban scale, quality-of-life index, annual health institution utilization rate, regional gross income per capita) characteristics are not enough to specify alone. Therefore, follow-up research should examine not only sports facilities but also factors affecting empirical sports participation and overcome the limitations of the connected components of individuals, families, and community.

## Figures and Tables

**Table 1:** Hierarchical regression analysis results.

Hierarchy Structure	Individual		Family		Community	
	b	β	b	β	b	β
Constant	5.627		5.458		4.295	
Sex (Female)	0.236	0.073	0.214	0.065	0.421	0.008 *
Age	0.320	0.148 **	0.304	0.148 **	0.202	0.146 ***
Subjective Health Status	1.114	0.202 ***	1.487	0.198 ***	4.759	0.168 ***
Household Members			−0.042	–0.029	–0.021	–0.013
Monthly Household Income			0.147	0.064	0.133	0.046
Urban scale (small- and medium-sized cities)					–0.388	–0.072 **
Urban scale (countryside)					–0.623	–0.179 ***
Quality-of-Life Index					5.218	0.482 ***
Annual Health Institution Utilization Rate					–0.971	–0.091
Regional Gross Income Per Capita					1.760	0.648 ***
*R*^2^ (Δ*R*^2^)	0.209		0.224 (0.015)		0.717 (0.507 ***)	
F	31.947 ***		47.372 ***		70.997 ***	

Note: * *p* < 0.05, ** *p* < 0.01, *** *p* < 0.001.

## Data Availability

Publicly available datasets were analyzed in this study. These datasets can be found here: (Korea Sports Promotion Foundation (kspo.or.kr accessed on 10 May 2021)), (KDCA).
